# Account of Diseases in an Eastern District of London, from the 20th of July, to the 20th of August

**Published:** 1799-10

**Authors:** 


### STATE OF DISEASES IN LONDON.

*Account of Diseases in an Eastern District of London, from the 20th of July, to the 20th of August.*

| | No. of Cases. |
|---|---|
| ACUTE DISEASES. | |
| Typhus Gravior | 2 |
| Typhus Mitior | 4 |
| Quotidian | 1 |
| Pneumonia | 3 |
| Catarrhus | 1 |
| Measles | 2 |
| Acute Rheumatism | 2 |
| CHRONIC DISEASES. | |
| Asthma | 4 |
| Cough | 12 |
| Dyspnœa | 9 |
| Phthisis Pulmonalis | 5 |
| Pleurodyne | 4 |
| Cephalæa | 4 |
| Epilepsia | 1 |
| Vertigo | 4 |
| Syncope | 3 |
| Palpitatio | 2 |
| Dyspepsia | 6 |
| Vomitus | 3 |
| Gastrodynia | 4 |
| Diarrhœa | 12 |
| Dysenteria | 4 |
| Colica | 3 |
| Colica Pictonum | 2 |
| Hæmorrhois | 3 |
| Dolor Nephriticus | 2 |
| Menorrhagia | 3 |
| Prolapsus Vaginæ | 1 |
| Dysmenorrhœa | 2 |
| Amenorrhœa | 4 |
| Cancer in Utero | 1 |
| Chlorosis | 5 |
| Dysuria | 4 |
| Enuresis | 2 |
| Hysteria | 3 |
| Scrophula | 4 |
| Herpes | 6 |
| Lichen | 1 |
| Psora | 1 |
| PUERPERAL DISEASES. | |
| Menorrhagia lochialis | 3 |
| Mastodynia | 6 |
| Ephemera | 3 |
| INFANTILE DISEASES. | |
| Hooping Cough | 4 |
| Measles | 5 |
| Aphthæ | 6 |
| Ophthalmia purulenta | 3 |

We may repeat the observation made in the last Report of the state of diseases, that notwithstanding the weather has been unusually cold and wet, the number of diseases has not been increased. Colds and coughs, indeed, have been rather more general than they are at this season of the year, owing probably to the sudden showers of rain which have fallen, and for which persons going abroad have not been prepared. Slight disorders of the bowels have still prevailed. Diarrhœas have been frequent, but of a mild and favourable kind, rather producing a salutary evacuation, than any morbid effect upon the constitution. Dysenteries have also occasionally occurred, accompanied with very slight degree of fever, and yielding pretty soon to the usual methods of cure. The measles have prevailed amongst children; but this, like the other diseases of the present season, has proved mild: the fever and cough, which are the symptoms of principal consequence in this disease, have been very slight.

☞ *For the other Lists of Diseases, see page 292.*

